# Detection of Ursid Gammaherpesvirus 2 in Asiatic Black Bears ( *Ursus thibetanus* ) With Keratoconjunctivitis

**DOI:** 10.1111/vop.70058

**Published:** 2025-08-15

**Authors:** Katharina Thieme, Marc Gölkel, J. Corinna Eule, Lesley Halter‐Gölkel, Dusan Kunec, Frank Göritz, Sina Feyer, Bich Ngoc Bui, Jakob Trimpert

**Affiliations:** ^1^ Unit for Ophthalmology, Centre for Veterinary Clinical Services Veterinary Hospital Freie Universität Berlin Berlin Germany; ^2^ Leibniz‐Institute for Zoo and Wildlife Research Berlin Germany; ^3^ Bear Sanctuary Ninh Binh Four Paws Viet Ninh Binh Vietnam; ^4^ Institute of Virology, School of Veterinary Medicine Freie Universität Berlin Berlin Germany; ^5^ Small Animal Clinic Veterinary Hospital Freie Universität Berlin Berlin Germany; ^6^ Department of Pathobiology and Diagnostic Medicine, College of Veterinary Medicine Kansas State University USA

**Keywords:** conjunctivitis, keratitis, ocular disease, ophthalmic, PCR, UrHV

## Abstract

**Objective:**

To investigate the potential contribution of herpesvirus infection in the development of ocular surface disease in Asiatic black bears ( *Ursus thibetanus* ).

**Animals Studied:**

Eight captive Asiatic black bears housed at a wildlife sanctuary were examined during routine health assessments.

**Methods:**

Fourteen cytobrush conjunctival samples were collected from eight subjects. A first bear was sampled in January 2024 with a pooled sample acquired from both eyes. Seven more animals underwent a full ophthalmic exam and conjunctival cytobrush sampling in February 2024. From those animals, one cytobrush sample per eye was acquired. DNA was extracted from the samples and tested for the presence of herpesviruses by polymerase chain reaction (PCR).

**Results:**

All but one animal showed evidence of active or past ocular surface disease. Nested PCR on a highly conserved region in herpesvirus genomes showed the presence of herpesvirus DNA in six samples obtained from 5 individuals. Three animals tested negative. PCR products from positive cases were subjected to Sanger sequencing, and results were aligned against the NCBI nucleotide collection. Sequences returned with over 95% similarity to Ursid gammaherpesvirus 2 (UrHV‐2). Five of the positively tested eyes (5/6, 83%) and two of the negatively tested eyes (2/9, 22%) showed active ocular surface disease.

**Conclusions:**

This study represents the first report of ursid gammaherpesvirus identification in bears with keratoconjunctivitis. The findings indicate a potential association between UrHV‐2 and active ocular surface disease in Asiatic black bears. Further research is warranted to clarify its role in the pathogenesis of ocular surface disease in this species.

## 1 Introduction

The Asiatic black bear ( *Ursus thibetanus* ), one of six species in the genus *Ursus* within the family Ursidae, is distributed across large parts of Southern and Eastern Asia. Over the past three decades, the global population of this species has declined by approximately 30%, with reductions exceeding 60% in Vietnam and Bangladesh (IUCN, 2020). Consequently, the Asiatic black bear is classified as vulnerable on the IUCN Red List [1]. Major threats to the species include habitat loss due to deforestation and agricultural expansion, compounded by hunting for their skins, paws, and gallbladders. The latter is driven by the demand for Traditional Asian medicine despite the availability of synthetic alternatives (ursodeoxycholic acid). Substantial numbers of Asiatic black bears, often captured from wild populations, are confined under conditions raising serious animal welfare concerns on farms across multiple countries, where they are subjected to repeated bile extraction. While bear bile farming remains legal in China, it has been outlawed in Vietnam since 2005, with governmental bear sanctuaries providing refuge and medical care for rescued individuals from bear bile farming and the illegal wildlife trade. However, health issues, limited safe habitat availability, and habituation to humans constrain the feasibility of reintroducing these animals into the wild.

Captive Asiatic black bears frequently suffer from a range of health conditions, including infections at bile extraction sites, abdominal hernias, peritonitis, cholecystitis, hepatic neoplasia, cardiovascular disease, skeletal abnormalities, and behavioral issues [2]. Additionally, ocular diseases are described as a recurrent concern in captive individuals [3].

In various species, infections with endemic α‐ and γ‐herpesviruses have been implicated in ocular surface diseases, such as keratoconjunctivitis and corneal ulcers [4, 5, 6, 7, 8, 9, 10].

Endemic herpesvirus infections in bears were first documented in 2013 when Lam et al. [11] identified a novel γ‐herpesvirus, Ursid herpesvirus 1 (UrHV‐1), in oral squamous cell carcinoma (SCC) lesions in sun bears ( *Helarctos malayanus* ). While a limbal SCC was observed in one bear, the involvement of UrHV‐1 in this ocular lesion was not specified. Subsequent studies revealed a high prevalence of UrHV‐1 infection in sun bears with oral SCC (70.6%) [12] and detected UrHV‐1 in 15.9% of a captive Bornean sun bear population [13]. Moreover, a gammaherpesvirus with 95% identity to UrHV‐1 was detected in black bears (*Ursus americanus*) with and without neurological signs [14].

Sequences of the glycoprotein B and DNA polymerase genes from putatively designated Ursid gammaherpesvirus 2 (UrHV‐2) and Ursid gammaherpesvirus 3 were detected in an Asiatic black bear ( *Ursus thibetanus* ) and a brown bear ( *Ursus arctos* ) in Russia and submitted to GenBank in 2018 (MK089801) and 2022 (OP751953), respectively. No descriptions of the associated findings were available for these entries at the time of this study. To the best of our knowledge, no other endemic herpesviruses have been described in ursids, and as of 2021, no ursid herpesvirus was formally accepted as a virus species by the International Committee for the Nomenclature of Viruses (ICTV) [15].

Ocular diseases such as conjunctival and corneal disorders, including indolent corneal ulceration resembling spontaneous chronic corneal epithelial defects observed in dogs, have been reported in captive Asiatic black bears [3, 16]. However, the potential role of herpesvirus in causing ocular surface disease in bears remains unexplored. This study investigated the potential role of herpesvirus infection as a contributing factor in the development of ocular surface disease in captive Asiatic black bears.

## 2 Materials and Methods

The sampling and analyses conducted in this study were part of routine clinical investigations addressing conditions observed in the animal population. These procedures were carried out by veterinarians responsible for managing the animals' health. All sampling and testing were performed to obtain diagnostic information relevant to the clinical condition and to inform future health management strategies. No sampling was performed for experimental or research‐specific purposes. Animal ethics approval was granted by the Animal Ethics Committee in Nong Lam University, Ho Chi Minh City, Vietnam (Permit No. AEC‐NLU241209).

Seven Asiatic black bears underwent routine health checks under general anesthesia in February 2024. The bears were induced in their indoor enclosure by blow pipe or hand injection with Tiletamine/Zolazepam (2.2 mg/kg, Zoletil 100, Virbac), Medetomidine (0.01 mg/kg, Medetin, Dong Bang Co. Ltd.), and Butorphanol (0.05 mg/kg, Butomidor, Richter Pharma) intramuscularly. After induction, all bears were intubated and transferred to the examination room. Anesthesia was maintained with sevoflurane (2,5%, Sevoflurane, Baxter) and medical oxygen (flow rates ranging from 2 to 5 L/min). Based on individual health status, examinations included a full physical exam, serum biochemistry and complete blood count, oral and dental exam, ultrasound examination of the heart and abdomen, radiography of the chest and other body parts as indicated, as well as bronchoscopy and gastroscopy for various laboratory diagnostic testing. All patients underwent a full ophthalmological exam, including slit‐lamp biomicroscopy (Kowa SL‐17, Kowa, Tokyo, Japan), indirect fundoscopy (Omega 100, Heine, Ettenheim, Germany) with a handheld 20D and 30D lens (Volk optical Inc., Ohio, USA), Schirmer tear test 1, rebound tonometry (Tonovet, Icare, Finland), gonioscopy (Koeppe goniolens 16 mm) and fluorescein staining. Cytobrush (Celltip cytobrush, Servoprax, Wesel, Germany) conjunctival samples were obtained from each eye of all bears. One additional bear was sampled in January 2024 without a complete ophthalmological exam being performed. The cytobrush samples were kept in dry tubes and were frozen immediately at −20°C until evaluation.

### 2.1 DNA Isolation

DNA was extracted from the conjunctival cytobrush samples using the innuPREP Virus DNA/RNA Kit (IST AG). Total extracted DNA was used as a template for nested PCR.

### 2.2 PCR Amplification and Sequencing

PCR targeted two regions that are highly conserved in herpesvirus genomes: the catalytic subunit of the DNA polymerase (panDPOL) and the conserved region of exon 2 of the ATPase subunit of the terminase gene (panCSG). The target sequences were amplified via nested PCR using degenerate primers, as previously described [17]. The primers used in this study are listed in Table 1.

**TABLE 1:** Primer sets used for amplification of DNA polymerase and terminase genes.

Gene	Primer	Primer sequence (5′‐3′)	Amplicon size (bp)
panDPOL	POLdeg1F	GAYTTYSMIAGYYTITAYCC	
	POLdeg1R	TTICKIACSARITCIACICCYTT	713–992
	POLdeg2F	ATIATIMWRGCICAYAAYYTITG	
	POLdeg2R	AAIAIISWRTCIGTRTCICCRTA	482–758
panCSG	CSGdeg1F	GTIGAYGARRSIMAYTTYAT	
	CSGdeg1R	TTKIIIGTRWAIGCIGGRTC	470–494
	CSGdeg2F	MYISYAARMTIATITTYRTITCITC	
	CSGdeg2R	GTRWAIGCIGGRTCIAIRTA	397–421

The nested PCR reactions were conducted under conditions similar to those previously described [17]. Briefly, 50 μL panDPOL PCR reactions contained 1 μM of each primer, 0.4 mM of each dNTP, 5% DMSO, and 5 U of DreamTaq Polymerase (Thermo Fisher Scientific). For the panCSG, the reactions contained 1 μM of each degenerate primer, 0.4 mM of each dNTP, 10% DMSO, 5% glycerol, and 5 U of DreamTaq Polymerase. For the first PCR, 5 μL of the total DNA was used as a template, and 2 μL of the primary PCR reaction was then used for the second, nested PCR.

The amplification conditions for the first panDPOL PCR were as follows: initial denaturation at 94°C for 2 min, followed by 40 cycles of 94°C for 30 s, 45°C for 3 min, and 72°C for 30 s, with a final extension at 72°C for 5 min. The second PCR was performed under the following conditions: initial denaturation at 94°C for 2 min, followed by 40 cycles of 94°C for 30 s, 40°C for 3 min, and 72°C for 30 s. The conditions for the amplification of the terminase gene were the same as for the DNA polymerase gene, except that the annealing temperature was 40°C in the first PCR and 35°C in the nested PCR.

PCR products were resolved by electrophoresis on a 2% agarose gel. The PCR products obtained with the nested POLdeg2 primers were purified from the agarose gel using the Monarch PCR & DNA Cleanup Kit (NEB) and were subsequently Sanger sequenced using the same primers.

### 2.3 Statistical Analysis

Statistical analysis was performed using IBM SPSS Statistics (Version 29). Comparisons between active and non‐active ocular surface disease in relation to UrHV‐2 PCR results were conducted using Fisher's exact test; a *p* value < 0.05 was considered statistically significant.

## 3 Results

All animals had been rescued from bear bile farms between 2017 and 2023. Age estimates were made upon arrival based on available background information and findings from their physical examinations. The precise age of the bears could not be determined; however, all individuals were estimated to be at least 20 years old, as they had been microchipped as adults by the government in 2005.

Details of the complete ophthalmic examination performed on seven of the eight bears are provided in the Data S1.

Apart from ocular disease, the main comorbidity diagnosed was chronic degenerative spinal disease in five individuals, which was treated with Gabapentin at 7–17 mg/kg twice daily (BID) per os (p.o.) and Meloxicam at 0.1 mg/kg once daily (SID) p.o. based on individual clinical appearance. Additionally, four bears suffered from chronic systemic hypertension and were treated with Enalapril at 0.5 mg/kg BID p.o. and Amlodipine at 0.1 mg/kg BID p. Two animals presented with seasonal alopecia of unknown origin, which resolved under Oclacitinib at 0.2 mg/kg SID p. Two other individuals presented with presumably stress‐associated chronic gastritis for which they received Omeprazole at 0.7 mg/kg SID p. Furthermore, one individual exhibited multiple abnormal repetitive behaviors, which were managed with Sertraline at 1.4 mg/kg SID p.o., while another bear repeatedly suffered from recurrent bacterial urogenital tract infection treated based on antibiograms and clinical symptoms.

In total, 14 cytobrush conjunctival samples were collected from the eight bears. Of these, one bear, which had previously undergone enucleation of one eye due to corneal perforation, was sampled only in the remaining eye. Another bear had both eyes sampled, with the samples pooled. The remaining six bears underwent separate sampling of both eyes.

**FIGURE 1:**
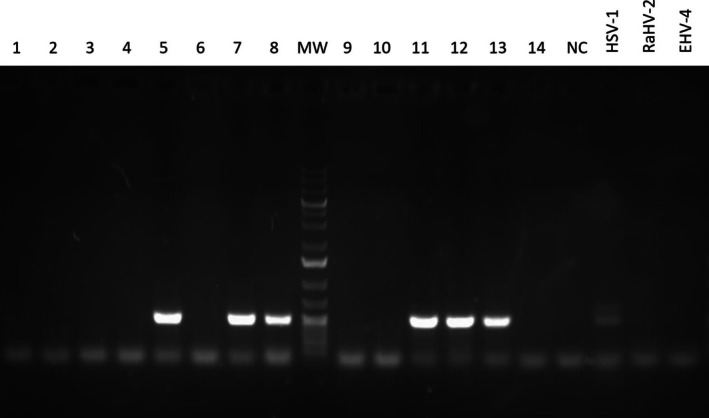
Agarose gel electrophoresis of PCR products obtained in the study. PCR amplification of a DNA polymerase gene with POLdeg2 primers is expected to yield a fragment of approximately 500 bp. The sample IDs are indicated above each lane. Additional controls included DNA from Herpes Simplex Virus 1 (HSV‐1), Ranid Herpesvirus 2 (RaHV‐2), and Equine Herpesvirus 4 (EHV‐4). MW = molecular weight marker (GeneRuler 1 Kb Plus DNA Ladder (Thermo Fischer Scientific)); NC = no template control.

PCRs performed with the nested POLdeg2 primers on samples numbered 5, 7, 8, 11, 12, and 13 produced specific products of expected size (approximately 500 bp, Figure 1). The PCR products were sequenced by Sanger sequencing, and the forward and reverse sequencing reads from these samples were assembled into a consensus sequence (see Data S2). The consensus sequence was then analyzed for nearest homology using the BLAST algorithm on the NCBI nucleotide database. The BLAST results showed that the obtained DNA polymerase sequences had over 95% similarity to Ursid gammaherpesvirus 2 (UrHV‐2), isolate BLB124, which contains a partial sequence of the DNA polymerase gene (GenBank: MK089801.1). The obtained sequence alignment is provided in the Data S2. The consensus sequence from PCR products obtained in this study was submitted to GenBank with the following accession number: PQ857575.

**FIGURE 2:**
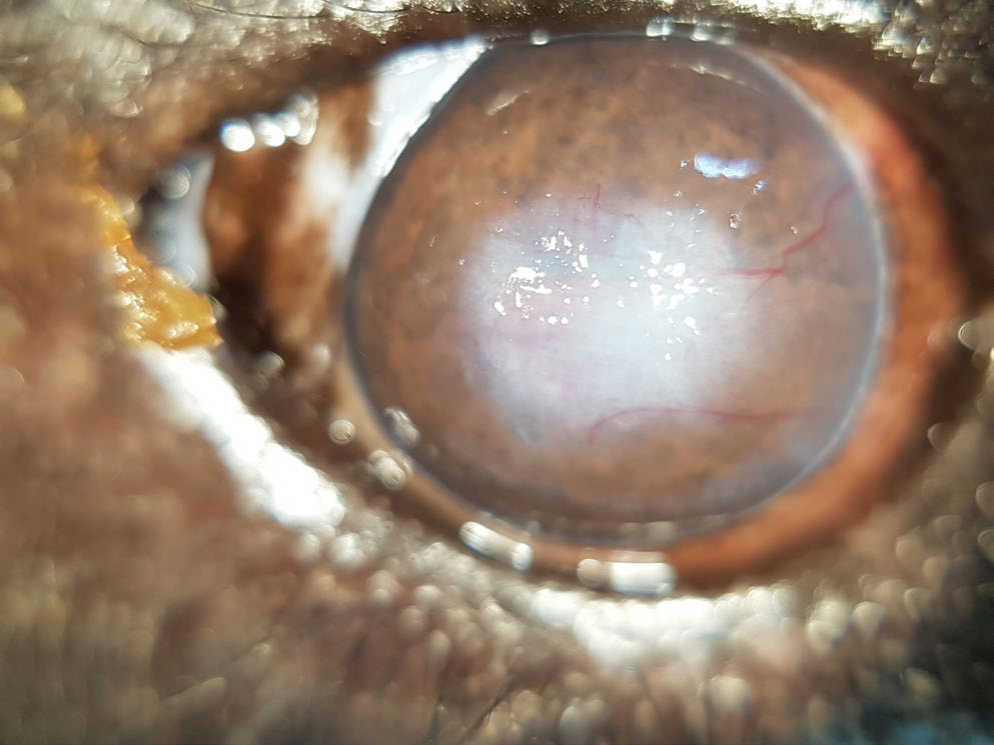
Right eye of an Asiatic black bear (Bear 8) showing chronic, active ocular surface disease. This case tested positive for UrHV‐2.

**FIGURE 3:**
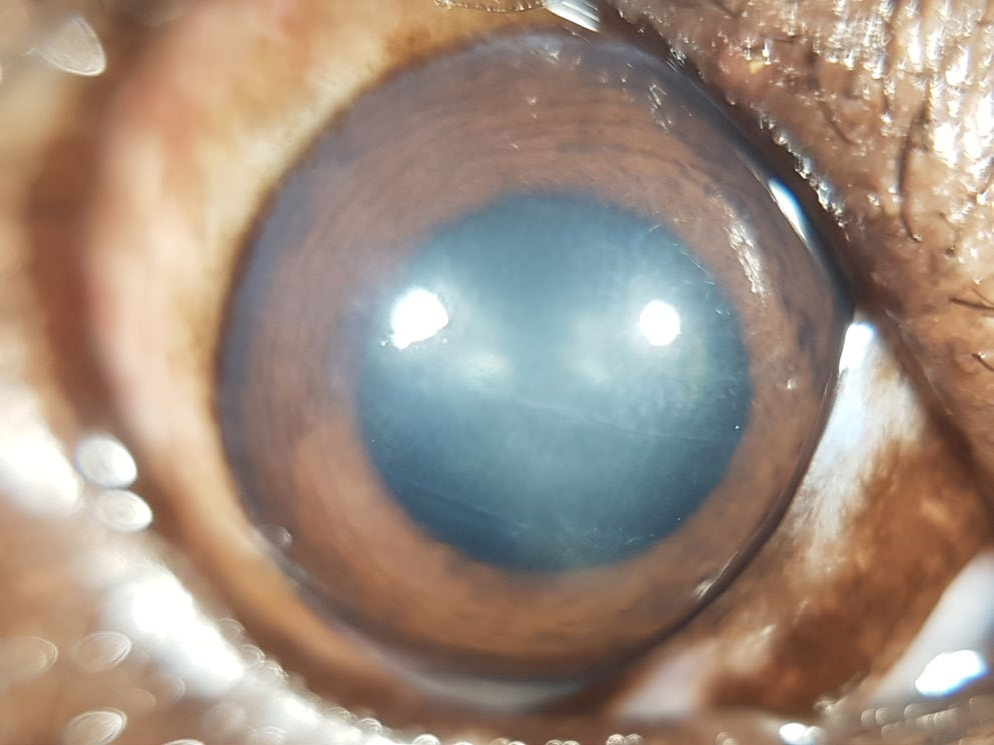
Right eye of an Asiatic black bear (Bear 3) exhibiting corneal scars and fibrosis resulting from a previous corneal disease. This eye tested negative for UrHV‐2.

All eyes of all bears that underwent a full ophthalmic exam had evidence of present (7/13) or past (6/13) ocular surface disease (Figures 2 and 3, respectively).

Of the positively tested eyes, all had active (5/6) or evidence of past (1/6) ocular surface disease. In four bears, one eye tested positive; and in one bear, both eyes tested positive for UrHV‐2 (Table 2).

**TABLE 2:** Summary of polymerase chain reaction (PCR) results for Ursid Gammaherpesvirus 2 and ocular surface findings of all bears.

	Sample	Eye	DNA PCR	Ocular surface findings
Bear 1	1	OS + OD	−	No abnormalities
Bear 2	2	OD	−	Corneal fibrosis, corneal mid‐stromal crystalline deposits, corneal perilimbal haze
	3	OS	−	Superficial, chronic, epithelial defect
Bear 3	4	OD	−	Corneal scars
	5	OS	+	Corneal scars
Bear 4	6	OD	−	Periocular alopecia, blepharitis, conjunctival hyperemia, conjunctival hyperplasia, corneal perilimbal haze
	7	OS	+	Periocular alopecia, blepharitis, mucopurulent ocular discharge, conjunctival hyperemia, conjunctival hyperplasia, corneal perilimbal haze
Bear 5	8	OD	+	Non‐ulcerative keratitis with corneal neovascularization, corneal perilimbal haze
Bear 6	9	OD	−	Conjunctival hyperplasia
	10	OS	−	Conjunctival hyperplasia
Bear 7	11	OD	+	Blepharitis, conjunctival hyperemia
	12	OS	+	Chemosis
Bear 8	13	OD	+	Superficial, chronic, epithelial defect
	14	OS	−	Corneal fibrosis with inactive corneal neovascularization, corneal perilimbal haze

*Note:* Samples testing positive for UrHV—2 are highlighted in gray.

Abbreviations: OD, right eye; OS, left eye.

Of the negatively tested eyes (*n* = 9), seven eyes of five bears had signs of active (2/7) or evidence of past (5/7) ocular surface disease. Both eyes of one bear (Bear 1) had no signs of ocular surface disease. However, this bear had not received a full ophthalmic exam. The ocular surface findings and PCR results of all bears are summarized in Table 2.

Eyes with active ocular surface disease were significantly more likely to test positive for UrHV‐2 (*p* = 0.041) than eyes without active ocular surface disease.

## 4 Discussion

To the authors' knowledge, this study is the first to report the detection of Ursid gammaherpesvirus 2 (UrHV‐2) in bears and the first to identify a herpesvirus from the ocular surface in this species. Sequences of the glycoprotein B and DNA polymerase genes from UrHV‐2 detected in an Asiatic black bear ( *Ursus thibetanus* ) in Russia were deposited in the GenBank database in 2018 (MK089801). However, no peer‐reviewed publication associated with these entries was available at the time of this study.

Although the prevalence of ocular surface disease in the studied population appeared high, the correlation with herpesvirus infection remains uncertain. Notably, clinical presentations of ocular lesions did not exhibit hallmark features typically associated with herpesvirus infections, such as punctate or dendritic ulceration. This lack of distinct presentation may reflect the chronicity of the observed conditions. Despite this, there was a statistically significant association between UrHV‐2 positivity and the presence of active ocular surface lesions, with 83% (5/6) of positive eyes affected by active disease compared to 22% (2/9) of negative eyes (*p* = 0.041). One bear that tested negative for UrHV‐2 did not undergo a complete ophthalmic examination, and minor pathologies could potentially have been overlooked. Qualified veterinarians routinely conduct thorough ophthalmic assessments of all bears during health checks, including visual evaluation of ocular surface structures, applanation tonometry, and direct ophthalmoscopy. While not formally trained in ophthalmology, all examiners have considerable experience in conducting ophthalmic examinations in bears. This expertise is confirmed by their selection of animals scheduled for a health check during the availability of an ophthalmologist, where even minor ocular conditions were presented.

The sampling strategy in this study was inherently biased, as it focused on animals presenting with ocular lesions during routine health evaluations coinciding with the on‐site availability of an ophthalmologist. As a result, the study design does not allow for definitive conclusions regarding the relationship between herpesvirus presence and ocular surface lesions. Future investigations that include samples from asymptomatic individuals could provide a broader understanding of herpesvirus prevalence within the population and clarify potential associations between ocular lesions and ursid gammaherpesvirus. Sampling could easily be performed during routine health checks, and asymptomatic animals could be used as a control group for future investigations. Additionally, until data on the prevalence of UrHV‐2 in wild Asian black bears becomes available, it remains unclear whether the infection is already ubiquitous within the population, or if captive conditions facilitate virus transmission, promote reactivation from latency, or influence its role in the development of ocular surface lesions. Obtaining the complete genome sequence of UrHV‐2, which remains unsequenced to date, should be prioritized in future research. A full genomic sequence would enhance the accuracy of virus detection and provide further insights into its role and impact on the health of bear populations.

This study's findings align with observations from equine gammaherpesvirus (EHV) research, where investigations into the relationship between EHV‐2 and EHV‐5 and ocular disease have yielded inconsistent results. Studies in horses have not consistently demonstrated a statistical association between EHV detection and ocular disease. Additionally, the high prevalence of EHV in horses without ocular pathology complicates the establishment of causality [5, 18, 19, 20]. In cats, the causality between feline alphaherpesvirus 1 infection and ocular surface disease is uncontroversial, yet there is still a high frequency of false‐positive and false‐negative results from PCR testing. Possible reasons include the intermittent viral shedding of infected animals, inadequate sample collection or storage, or clinically healthy animals that shed the virus in response to stress [7]. Additionally, herpesvirus reactivation can be triggered by corneal nerve disruption or anti‐dromic stimulation, as shown in animal models [21]. This makes it difficult to determine whether the virus is a causative agent of corneal ulceration or reactivates as a consequence of it. Generally, the specific biology of herpesviruses, with their ability to establish life‐long latency after initial infection and reactivate in response to a variety of factors, complicates diagnostics in this family of viruses [22]. In the absence of a virus isolate and suitable cell culture models of UrHV‐2 infection, our ability to study the biology of this relatively novel herpesvirus is limited. An in vitro study examining the effects of the virus on corneal and conjunctival cell cultures could help determine whether it has cytopathic effects in these tissues, thereby providing further support for its potential role in keratoconjunctivitis in bears. Therefore, future efforts need to include attempts to isolate the replicating virus from infected bears and identify cell cultures permissive to UrHV‐2 replication.

Wildlife species often endure elevated stress levels in captive environments, which can result in persistent adrenocortical activity. Such prolonged stress responses may influence various physiological processes, including the regulation of the immune system [23]. This interplay between stress and immune function is particularly relevant to the behavior of herpesviruses, which exhibit latency as a defining characteristic. Latency involves complex modifications of the host's immune responses, allowing the virus to remain largely undetected within infected cells. Although the specific site of latency for certain gammaherpesviruses remains unclear, these viruses are known for their pronounced lymphotropism. Memory B‐cells and germinal centers, in particular in the spleen and tonsils, have been suggested as a potential site of latency [24]. Supporting this hypothesis, Officer et al. [12] reported a high prevalence of UrHV‐1 in the tonsils of sun bears.

Studies have demonstrated a reduction in overall stress levels post‐rescue in bears rescued from bear bile farming [25]. However, these bears also exhibited increased fecal cortisol metabolites in response to certain management interventions. Notably, some peaks in fecal cortisol metabolites were observed in the absence of identifiable stressors, suggesting potential complexities in stress responses. Bears are predominantly solitary in the wild [26]. This behavioral characteristic contrasts with the conditions observed in rescue centers, where the animals are housed in much closer proximity than they would experience in the wild. While these settings are often unavoidable, they may serve as a potential source of stress for the bears, as assumed to be the case in two of the sampled individuals presenting with chronic gastritis. Moreover, such close physical contact among individuals can significantly increase the risk of disease transmission, highlighting the need for management strategies.

The *Miscanthus* sp., also known as Chinese silver grass or Elephant grass, is a potential source of corneal injury potentially contributing to the high prevalence of ocular surface lesions in this bear population. This plant covers large areas of the enclosures at the rescue center and is challenging to eradicate due to its fast growth and resilience. The leaves of *Miscanthus* possess sharp edges with a saw‐tooth pattern when viewed under the microscope. Similar concerns have been reported in other species. *Miscanthus* bedding was implicated in initial corneal injuries in red‐legged partridges, ultimately leading to mycotic keratoconjunctivitis [27].

The topical treatment of ocular conditions in bears presents significant challenges; however, advancements in sustained‐release drug delivery technologies offer promising solutions. Innovative approaches such as episcleral implants [28], nanoparticle delivery systems [29], prodrugs, peptides, and in situ gelling formulations [30] continue to expand therapeutic options.

Currently, investigations into effective antiviral treatments for UrHV‐2 are lacking. Nonetheless, the demonstrated efficacy of idoxuridine and cidofovir in treating gammaherpesvirus infections of the ocular surface in other species suggests their potential for managing ocular herpesviral infections in bears [31]. Further research is necessary to evaluate these antivirals' efficacy and safety specifically in ursine patients.

Ideally, comprehensive testing for the presence of UrHV‐2 should be conducted across the entire population of the bear sanctuary. Bears testing positive for the virus could then be housed separately from negative individuals to minimize the risk of transmission. Similarly, newly arriving bears should be screened for UrHV‐2 prior to integration into the existing population, with housing arrangements made based on their infection status. Consideration should also be given to the UrHV‐2 status of individuals planned for release into the wild, as releasing infected bears could potentially introduce the virus to wild populations, with unknown ecological consequences. However, practical challenges such as limited space, the potential disruption of established social structures, and the currently unknown impact of the virus on the health of infected individuals render the implementation of such measures impractical and unjustified at this time. Further research is needed to evaluate the prevalence and health implications of UrHV‐2 infection in captive and wild bears before interventions are considered.

This study highlights the need for ongoing health surveillance in both captive and wild Asiatic black bears to better understand the prevalence of UrHV‐2 and its impact on ocular health. Such information is essential for rehabilitation centers to perform informed risk assessments regarding the management, housing, and potential future release of these bears. Additionally, these findings would contribute significantly to the development of more effective conservation strategies and management plans, benefiting organizations dedicated to Asiatic black bear conservation efforts.

## 5 Limitations

The present study has several limitations. First, the small sample size restricts the conclusions that can be drawn about the sanctuary population and limits extrapolation to a broader population of captive or wild bears. Second, the absence of a control group, such as bears without ocular lesions or known UrHV‐2 infection, hinders the ability to establish definitive causal relationships between the virus and observed clinical manifestations. Notably, only one bear without ocular surface abnormalities was included in the study; while it tested negative for UrHV‐2, no meaningful conclusions can be derived from this single case. A well‐defined control group would provide a critical baseline for comparison, strengthening the reliability of the study's conclusions. Lastly, the absence of virus isolation limits the ability to confirm active infection. Although molecular detection methods such as PCR can identify the presence of viral DNA, they cannot differentiate between latent, inactive, or actively replicating states of the virus. While not possible in this study, transcriptome analysis of herpesvirus infection can be employed to differentiate between lytic and latent stages of viral infection. Lytic replication is characterized by the expression of numerous viral transcripts, while only very few loci are transcribed during latency [32]. This technology could help elucidate the nature of UrHV‐2 in cases such as those described here. Addressing these limitations in future studies through larger sample sizes, the inclusion of appropriate control groups, and the implementation of viral isolation techniques will be essential to gaining a more comprehensive understanding of UrHV's role in ocular health in bears.

## 6 Conclusion

In conclusion, this is the first report of UrHV‐2 in the Asiatic black bear. This report suggests that UrHV‐2 may be associated with ocular surface disease in this species. The prevalence of this virus in the captive and wild Asiatic black bear populations, as well as its pathogenicity and relationship to ocular surface disease, is unknown and warrants further investigation.

## Author Contributions

**Katharina Thieme:** conceptualization, data curation, formal analysis, methodology, investigation, writing – original draft, writing – review and editing, project administration, supervision. **Marc Gölkel:** conceptualization, data curation, methodology, investigation, project administration, writing – review and editing. **J. Corinna Eule:** conceptualization, resources, supervision, writing – review and editing. **Lesley Halter‐Gölkel:** writing – review and editing, investigation, data curation. **Dusan Kunec:** writing – review and editing, investigation. **Frank Göritz:** resources, writing – review and editing, validation. **Sina Feyer:** writing – review and editing, investigation. **Bich Ngoc Bui:** writing – review and editing, investigation. **Jakob Trimpert:** writing – review and editing, writing – original draft, conceptualization, resources, supervision, data curation, investigation, formal analysis, validation.

## Ethics Statement

This study was approved by the Animal Ethics Committee at Nong Lam University, Ho Chi Minh City, Vietnam (Permit No. AEC‐NLU241209). Animals were not captured, restrained, sedated, or anesthetized solely for this study.

## Conflicts of Interest

The authors declare no conflicts of interest.

## Supporting information


**Data S1.** Supporting Information.

**Data S2.** Supporting Information.

## Data Availability

The data that supports the findings of this study are available in the Supporting Information of this article.
