# Supplementary material for: Detection of Ursid Gammaherpesvirus 2 in Asiatic Black Bears ( Ursus thibetanus ) With Keratoconjunctivitis
Source: Vet Ophthalmol. 2025 Aug 15;29(2):e70058. doi: 10.1111/vop.70058 (PMC12951278; doi:10.1111/vop.70058)
Supplement: Supplementary file 1 — Data S1. Supporting Information. [file VOP-29-0-s002.docx]

Table summarizing the findings of the ocular examination of the bears undergoing a full ophthalmic exam during routine health checks under general anesthesia in February 2024.

|  | Ocular examination findings | | STT | | IOP | | UrHV-2 | |
| --- | --- | --- | --- | --- | --- | --- | --- | --- |
|  | OS | OD | OS | OD | OS | OD | OS | OD |
| Bear 2 | slight purulent discharge; lids: w.n.l.; conjunctiva: w.n.l.; cornea: lackluster appearance, irregular surface, fibrosis and edema between 9 and 6 o’clock, arborizing superficial neovascularization, fluorescein-positive spot at 10 o’clock, 1 mm in diameter; AC: w.n.l.; iris: w.n.l.; pupil: w.n.l.; lens, vitreous and fundus not visible due to corneal opacity; gonioscopy: not possible due to corneal opacity | no discharge; lids: w.n.l.; conjunctiva: w.n.l.; cornea: dorso-central fibrosis between 10 and 2 o’clock, mid-stromal faint crystalline deposits, faint perilimbal corneal haze, fluorescein-negative, AC: w.n.l.; iris: w.n.l.; pupil: w.n.l.; lens: nuclear sclerosis; vitreous: asteroid hyalosis, moderate syneresis; fundus: ONH w.n.l; multifocal bullous RD, beading of retinal vessels, hyperpigmented spot at 2 o’clock; gonioscopy: w.n.l. | 6 | 9 | 25 | 20 | - | - |
| Bear 3 | no discharge; lids: w.n.l.; conjunctiva: w.n.l.; cornea: smooth specular reflection, edema and fibrosis centrally extending peripherally between 1 and 3 o’clock, ghost vessels at 12 and 3 o’clock, no infiltrates, no deposits, fluorescein-negative, AC: w.n.l.; iris: w.n.l., pupil: w.n.l.; lens: nuclear sclerosis; vitreous: w.n.l.; fundus: slight generalized hyperreflectivity, attenuated retinal vessels, ONH w.n.l., ventrally three hyperpigmented lesions;  gonioscopy: ciliary cleft open, pectinate ligaments look like a dense web with fibrae latae and flow holes | no discharge; blocked meibomian gland mid-upper eyelid; conjunctiva: w.n.l., plant foreign body in the medial canthus; cornea: central fibrosis, two horizontal scars in the axial and ventral cornea, fluorescein-negative; AC: w.n.l.; iris w.n.l.; pupil w.n.l.; lens: nuclear sclerosis; vitreous: w.n.l.; fundus: ONH w.n.l., multifocal, well-demarcated hyperpigmented lesions close to ONH ventrally, medially and dorsally, slight, generalized hyperreflectivity, attenuated retinal vessels; gonioscopy: ciliary cleft open, pectinate ligaments look like a dense web with fibrae latae and flow holes | 9 | 8 | 23 | 23 | + | - |
| Bear 4 | purulent discharge; lids: irregular mucocutaneous junction, mid-ventral lower lid notch with accompanying small mass, conjunctiva: hyperplastic in the region of the lateral and medial canthus, at medial canthus conjunctiva in folds and hyperpigmented, no follicles; cornea: slight perilimbal haze; AC: w.n.l.; iris w.n.l., slight depigmentation peripherally, lens: nuclear sclerosis, cortical cataract between 10 and 11 o’clock peripherally, nebulous appearance, vitreous: w.n.l.; fundus: w.n.l.; gonioscopy: w.n.l. | no discharge; lids: irregular mucocutaneous junction, conjunctiva: hyperplastic, in the region of the medial canthus conjunctiva in folds and hyperpigmented, no follicles; cornea: slight perilimbal haze; AC: w.n.l.; iris w.n.l., slight depigmentation peripherally, lens: nuclear sclerosis, vitreous: w.n.l.; fundus: w.n.l.; gonioscopy: w.n.l. | 9 | 13 | 21 | 18 | + | - |
| Bear 5 | enucleated | no discharge; lids w.n.l.; conjunctiva: w.n.l.; cornea: irregular with fibrosis and edema axially, fluorescein-negative, inactive mid-stromal neovascularization starting dorsally, slight perilimbal haze; AC: w.n.l.; iris: small unpigmented mass peripheral at 10 o’clock; lens: nuclear sclerosis; vitreous: w.n.l.; fundus: pale ONH, generalized moderate hyperreflectivity with extensive regions of severe hyperreflectivity dorsotemporally, ventrally and medially to ONH, multifocal small retinal hemorrhages along the retinal vessels with surrounding edema, retinal vasculature attenuated; gonioscopy: w.n.l. | n.a. | 9 | n.a. | 19 | n.a. | + |
| Bear 6 | no discharge; lids: w.n.l.; conjunctiva: w.n.l.; free edge of nictitating membrane with slight follicles, slight conjunctival hyperplasia; cornea: w.n.l.; fluorescein-negative; AC: w.n.l.; lens: nuclear sclerosis, axially single anterior cortical punctate cataract; vitreous w.n.l.; fundus: beading in a single retinal vein, gonioscopy: w.n.l. | no discharge; lids: upper eyelid with healed scar; conjunctiva: missing upper punctum, free edge of nictitating membrane with slight conjunctival hyperplasia; cornea w.n.l.; fluorescein-negative; AC w.n.l.; lens: nuclear sclerosis, axially single anterior cortical punctate cataract; vitreous: w.n.l.; fundus: w.n.l.; gonioscopy: w.n.l. | 13 | 13 | 19 | 21 | - | - |
| Bear 7 | no discharge; lids w.n.l., small palpebral fissure; conjunctiva: slight swelling, no hyperemia, missing upper punctum, lower punctum small, cornea w.n.l.; fluorescein-negative; AC: w.n.l.; iris: iris hypoplasia; lens: small, axial, triangular, anterior cortical cataract, nuclear sclerosis; vitreous: w.n.l.; fundus: white exudate over and ventromedial to ONH, ONH itself w.n.l.; dorsolaterally at 3 and 4 o'clock retinal edema punctate retinal hemorrhage at 3 o’clock; gonioscopy: palpebral fissure too small to accommodate goniolens | serous discharge, periocular alopecia, lids: irregular mucocutaneous junction, meibomian gland openings small and few in number, small lacrimal puncta, high tear meniscus; conjunctiva: moderately swollen, no hyperemia, formed lymph follicles; cornea w.n.l.; AC w.n.l.; iris: iris hypoplasia: lens: nuclear sclerosis; vitreous w.n.l.; fundus: bullous RD around ONH, multiple punctate retinal hemorrhages; gonioscopy: palpebral fissure too small to accommodate goniolens | 13 | 17 | 28 | 25 | + | + |
| Bear 8 | no discharge; lids w.n.l.; conjunctiva: w.n.l.; cornea: slight perilimbal haze, axial and temporal fibrosis with corresponding inactive neovascularization from 3 o’clock extending axially, even corneal surface with normal specular reflection, fluorescein-negative; AC: w.n.l.; pupil: w.n.l.; lens: nuclear sclerosis; vitreous w.n.l.; fundus: two spots of peripapillary retinal edema with small central hemorrhage, punctate hemorrhage at 1 o’clock; gonioscopy: w.n.l. | squinting noted in the den; no discharge; lids: w.n.l.; conjunctiva: w.n.l.; cornea: slight perilimbal haze, axial fibrosis and edema with midstromal neovascularization temporally, axial cornea uneven with scattered specular reflection, moderate edema surrounding the vessels, suspected puncture channel with cellular infiltrate (<1mm), epithelium closed, fluorescein-negative, AC: w.n.l.; pupil: w.n.l.; lens: nuclear sclerosis; vitreous: w.n.l.; fundus: small area of peripapillary edema, gonioscopy: w.n.l. | 8 | 8 | 23 | 25 | - | + |

Abbreviations: OS, oculus sinister, left eye; OD, oculus dexter, right eye; STT, Schirmer-Tear-Test; IOP, intraocular pressure; UrHV-2, results of PCR testing for UrHV-2; w. n. l., within normal limits; AC, anterior chamber; ONH, optic nerve head; RD, retinal detachment
