# Supplementary material for: Detection of Ursid Gammaherpesvirus 2 in Asiatic Black Bears ( Ursus thibetanus ) With Keratoconjunctivitis
Source: Vet Ophthalmol. 2025 Aug 15;29(2):e70058. doi: 10.1111/vop.70058 (PMC12951278; doi:10.1111/vop.70058)
Supplement: Supplementary file 2 — Data S2. Supporting Information. [file VOP-29-0-s001.docx]

- **Assembled partial sequence of the DNA Pol gene from Ursid gammaherpesvirus 2**

GCGCATAACCTGTGCTACTCCACCTTAATCCTCGATGAAAATCTACACAGGTACCCTCACCTCAAAAACACAGACTATGAGACCTTCTATATCAGCTCCGGGCCCATCCACTTTGTTAAAACCCACGTGACAGAATCACTCCTAGGAAAGCTGCTAAAGATATGGCTAGCCAAGAGAAAGGCCATCAAAAAGCAACTGGAACAGTGTGCAGACCCAGTTCAAAAAACCCTCCTTGATAAACAACAGTTGGCCATCAAGGTTACCTGCAACTCTGTGTATGGGTTCACCGGGGTTGCTACCGGCCTATTTCCCTGTGTGAAAATAGCTGAGACAGTAACGCTCCAGGGACGTACCATGCTGGAGAAATCAAGGGCCTTCATTGAGGCAATCACGGCGGACCAGCTCTCGGTCATAACAAGAAGAGAGTTGGCCTTCGGCCCCGGGAGCGAAGTTCAGAGTCATTTACGGCAGACACCGA

- **BLAST Alignment Result**

The DNA Pol sequence obtained in this study (query) was aligned with the GenBank sequence MK089801.1 (Ursid gammaherpesvirus 2 isolate BLB124 glycoprotein B and DNA polymerase genes, partial cds) using the BLASTn algorithm.

| **Score** | **Expect** | **Identities** | **Gaps** | **Strand** |
| --- | --- | --- | --- | --- |
| 837 bits(453) | 0.0 | 460/463(99%) | 1/463(0%) | Plus/Plus |

Query 1 GCGCATAACCTGTGCTACTCCACCTTAATCCTCGATGAAAATCTACACAGGTACCCTCAC 60

|| |||||||| ||||||||||||||||||||||||||||||||||||||||||||||||

Sbjct 3221 GCTCATAACCTCTGCTACTCCACCTTAATCCTCGATGAAAATCTACACAGGTACCCTCAC 3280

Query 61 CTCAAAAACACAGACTATGAGACCTTCTATATCAGCTCCGGGCCCATCCACTTTGTTAAA 120

||||||||||||||||||||||||||||||||||||||||||||||||||||||||||||

Sbjct 3281 CTCAAAAACACAGACTATGAGACCTTCTATATCAGCTCCGGGCCCATCCACTTTGTTAAA 3340

Query 121 ACCCACGTGACAGAATCACTCCTAGGAAAGCTGCTAAAGATATGGCTAGCCAAGAGAAAG 180

||||||||||||||||||||||||||||||||||||||||||||||||||||||||||||

Sbjct 3341 ACCCACGTGACAGAATCACTCCTAGGAAAGCTGCTAAAGATATGGCTAGCCAAGAGAAAG 3400

Query 181 GCCATCAAAAAGCAACTGGAACAGTGTGCAGACCCAGTTCAAAAAACCCTCCTTGATAAA 240

||||||||||||||||||||||||||||||||||||||||||||||||||||||||||||

Sbjct 3401 GCCATCAAAAAGCAACTGGAACAGTGTGCAGACCCAGTTCAAAAAACCCTCCTTGATAAA 3460

Query 241 CAACAGTTGGCCATCAAGGTTACCTGCAACTCTGTGTATGGGTTCACCGGGGTTGCTACC 300

||||||||||||||||||||||||||||||||||||||||||||||||||||||||||||

Sbjct 3461 CAACAGTTGGCCATCAAGGTTACCTGCAACTCTGTGTATGGGTTCACCGGGGTTGCTACC 3520

Query 301 GGCCTATTTCCCTGTGTGAAAATAGCTGAGACAGTAACGCTCCAGGGACGTACCATGCTG 360

||||||||||||||||||||||||||||||||||||||||||||||||||||||||||||

Sbjct 3521 GGCCTATTTCCCTGTGTGAAAATAGCTGAGACAGTAACGCTCCAGGGACGTACCATGCTG 3580

Query 361 GAGAAATCAAGGGCCTTCATTGAGGCAATCACGGCGGACCAGCTCTCGGTCATAACAAGA 420

||||||||||||||||||||||||||||||||||||||||||||||||||||||||||||

Sbjct 3581 GAGAAATCAAGGGCCTTCATTGAGGCAATCACGGCGGACCAGCTCTCGGTCATAACAAGA 3640

Query 421 AGAGAGTTGGCCTTCGGCCCCGGGAGCGAAGTTCAGAGTCATT 463

|||||||||||| ||||||||||||||||||||||||||||||

Sbjct 3641 AGAGAGTTGGCC-TCGGCCCCGGGAGCGAAGTTCAGAGTCATT 3682
